# Contributions to Operative and Mechanical Dentistry

**Published:** 1842-12

**Authors:** W. H. Elliot

**Affiliations:** Member of the American Society of Dental Surgeons.


### ARTICLE III.

*Contributions to Operative and Mechanical Dentistry.* By W. H. Elliot, Member of the American Society of Dental Surgeons.

No. 1.

### ON FILLING TEETH.

Notwithstanding much has been written on the subject of filling teeth, yet less time and thought are generally devoted to it, than the importance of this branch of the dental science demands. Simple as it may appear, it is still a subject of interest, affording several highly instructive and interesting experiments.

Differing in views on filling from many persons who have grown grey in the practice of dental surgery—gentlemen for whose opinions I shall ever entertain the highest respect, I feel it incumbent upon me to be as unpretending in the exposition of my principles of practice, as the nature of the case will admit, leaving it to those who are able to understand the subject better, to draw their own conclusions from the facts here presented.

Contrary to the universally received opinion that cavities in teeth should be excavated larger within than at the opening, I advance the doctrine, that in, by far, the greater number of cases they should be made *quite as large in circumference at the orifice as at any other part, and in very many cases larger.*

The form which should be given to the cavity, I conceive, depends—1st, upon the liability of disturbance to the filling by any accident that may occur during the process of mastication. 2nd, Upon its depth compared with its circumference. 3d, Upon the facility with which the filling may be placed within it.

It often happens, while eating hard substances, that a single filling alone sustains the whole force of the masticating process. This occurs to such situated on the grinding surfaces of the teeth. Where it does occur, if the force be greater than that given it by the hand, which placed it there, the plug is necessarily driven further into the cavity, causing it to appear as having lost a part of its substance. Now if the cavity be *smaller at the bottom than at the orifice,* mechanical force, coming from any direction, (unless indeed it be in one more oblique than the walls of the cavity,) will only serve to consolidate the filling. But if the cavity have the same circumference at its floor as at its entrance, mechanical force applied out of the vertical, will most certainly drive the plug from one side of the cavity and press it against the opposite. If it be larger at the bottom than at the orifice, that part of the plug which once occupied the narrower is now driven to a broader part of the cavity, consequently a crevice is left between it and the substance of the tooth. Though this space may not always be visible to the naked eye, nevertheless it *always* exists, occupying a part, if not the whole, of the circumference; and if the cavity be considerably larger within, the filling is forced directly away from that portion of the tooth surrounding the orifice, thereby forming a vacuum, which is no sooner produced than it is liable to fill, with any fluid, that may happen to be in the mouth. This may follow not only accidental mechanical force applied, but the last efforts of the operator to consolidate the filling may produce it.

Here I anticipate the argument, that the expansion of the filling in all directions is sufficient to keep it in contact with the inner surface of the walls of the cavity, and here to, I will adduce a simple fact, which is worth a hundred arguments. Actual experiment proves in every case, that the fillings in use do not expand sufficiently, in all directions, to keep them in contact with all parts of the cavity, however compressible may be our material for filling.

It is sometimes impracticable to open the orifice equal in extent with the inner parts of the cavity; such cavities, being found on the grinding surfaces of the teeth may be filled with more compressible materials, tin or lead; and in such cases, the pointed plugger can be used to advantage.

Experiments proves also, that it requires double the force to sink the filling a certain distance in a cavity with an orifice larger than the bottom, that it does to sink it the same distance in one having an orifice smaller. I mean cavities of the same capacity, filled as near alike as possible. The reason is, one cavity is *honestly* full, while the other only appears to be so.

In answer to the argument that there is nothing to hold the filling in, I will only state, that there is nothing to throw it out.

I acknowledge when disease once gets under a filling, largest on the external surface, (to which however it is less liable,) it is much more readily thrown out. This is one of the advantages of the principle. In the other case, on the contrary, if the plug be retained in the cavity, decay goes on *secretly,* until the destruction of the nerve, if not of the whole tooth is effected.

Here let me assure my professional brethren, that they have much less to do with fastening the filling in the cavity, than is generally acknowledged. That is merely a *secondary* object. The first great point to be attained, is to introduce the foil in such a manner, that even the most subtle fluid cannot be forced in between it and the structure of the tooth. This accomplished, there will be little danger of losing the filling, unless by unavoidable accident.

It is a well ascertained fact, that the loss of nine fillings out of ten, is owing to a change having taken place in the condition of the bone of the tooth, (that is, if the tooth had been freed from disease and inflammation at the time the operation was performed,) which derangement in almost every case is chargeable to imperfections in the packing of the fillings.

In cavities smaller at the bottom, the angle formed by the internal and external surface of the tooth, is obtuse, and on that account, the enamel, being supported by a firm and healthy bone, seldom crumbles around the plug, and never to a very great extent. But in cases where the cavity has the opposite shape, the enamel immediately around the filling, having nothing to support it, is easily broken down. In the former case, that part of the filling which comes in contact with the tooth, is on examination, usually found denser than the interior of the plug; but in the latter, the filling is never found perfectly packed, except at the floor of the cavity, and at the external surface. Now when the edge of the enamel crumbles away, free ingress to all parts of the cavity, is given to the fluids of the mouth, and consequently a diseased condition of the bone supervenes. This is the true *pathological* cause of the loss of the plug, and not merely because it has lost its *mechanical hold* upon the tooth.

Here I will advert to to the very unscientific practice of thrusting the foil about, vainly endeavoring to pack it first in one part of the cavity and then in another. The fallacy of this method is most satisfactorily shown, by making a cavity with a small orifice in the end of a thick glass tube, in which it will be seen that in every attempt to force the foil against one side of the cavity, it is drawn away from the opposite.

In case a molar or bicuspid be decayed on its lateral surface, and that decay advanced so far as to involve the grinding surface of the tooth, the prominent parts around the cavity may be filed away until the orifice shall be level or unbroken, and occupying a position more or less oblique with the grinding surface. The sides of the cavity may now be cut down as near as possible at right angles with the plane of the orifice. This manner of filing adds much to the facility of performing the operation, and consequently to the firmness of the filling.

The greater number of the cases in which it becomes necessary to excavate the cavity larger at the bottom than at the orifice, are found on the lateral surfaces of the incisors, occupying nearly the whole length and width of the crown, having a depth equal to not more than one-third of its width, or one-fifth of its length. In such cases the upper part of the cavity is to be filled first, forcing the first layer in a directon upward against the upper wall, and inward against the bottom, and so on, adding layer after layer in plaits, until the cavity be full, taking care to leave as much of each layer out of the cavity as is practicable. With a broad pointed filling instrument and a file, the plug may now be brought to a level with the orifice. In this case, it will be remembered that the layers were placed in the cavity at right angles, or nearly so, with the plane of the orifice; in which position foil expands much more readily on the application of the instrument, than in any other.

I would not be understood as saying, that all cavities found on the lateral surfaces of the incisors are to be treated in this manner. If they have a depth equal in extent with their shortest diameter, they should by no means be excavated larger at the bottom than at the opening. If a cavity be found on the neck of a tooth, having a depth not more than equal to one-half of its diameter, it may be made a very little larger within, than at the orifice. In such cases, the filling instruments invented by Dr. Hayden are pre-eminently useful.

There remains another point in which my practice of filling differs from that of other dentists; and that is the preparation of the foil previous to placing it in the tooth. I cut from the leaf a strip of sufficient width to fill the cavity, then gather it into fine gathers like a cap ruff, making the plaits with one hand, and crushing them between the thumb and fingers of the other, until the whole piece be gathered, then straighten it out carefully, and gather again. This gives the foil a perfectly broken surface. The leaf may now be made into a roll a little larger in diameter than the mouth of the cavity. The yielding softness with which foil prepared in this manner gives way before the filling instrument, is sufficient proof of its superiority to all other methods. Experiment proves that it fills the inequalities of the bone to a greater degree of perfection than foil rolled or folded.

The roll when used is to be laid across the orifice in every direction. The force should always be given in a direction through the centre of the cavity, confining the instrument as much as possible to the centre of the orifice. When it becomes necessary to press the sides of the plug, an instrument having a point as large as the orifice may be chosen, and the whole surface forced down at once.*

* The foregoing article contains some good practical views on the filling and filing of teeth, but we think the author has fallen into some errors; one on the originality of his views on the best form the cavity should take. There are few dentists who have much experience, who are not aware of the necessity of making the cavity the same or nearly the same size at the orifice as at the bottom.* The latter is the best in most cases. We see no advantage in having the bottom of the cavity, under any circumstances, smaller than the orifice, for one of the above form can always be completely filled and firmly compressed with suitable instruments. The cases referred to by our author, in which he speaks of "mechanical force applied out of the vertical will most certainly drive the plug from one side, and press against the opposite," apply only to those cases where the cavity has not been completely filled and the metal well compacted. We are not sure we understand what our author means by "the expansion of the filling in all directions," but if he means to say that gold or foil cannot be so pressed into a cavity of the form above recommended, as to prevent their being still more compressed in mastication, so as to leave a "vacuum" for the admission of saliva, &c., we must beg leave to differ. His reasoning and arguments apply with force against small orifices and large internal cavities. We have never tried the plan recommended, of gathering or crimping the foil preparatory to insertion, but it appears from general principles liable to the objection of hardening the metal. On the whole we commend this article to the attentive perusal of the student. M.

* See Bell and Harris, also Parmly's introductory address in No. 1, vol. iii.

